# N-terminal pro-brain natriuretic peptide and adverse outcomes in Chinese patients with hypertrophic cardiomyopathy

**DOI:** 10.1042/BSR20212098

**Published:** 2022-01-06

**Authors:** Zeng-Lei Zhang, Yan-Yan Xu, Zhen Qin, Yong-Zheng Lu, Tian-Ding Liu, Li Zhang, Jia-Hong Shangguan, Wei Wang, Jun-Nan Tang, Jin-Ying Zhang

**Affiliations:** 1 Department of Cardiology, First Affiliated Hospital of Zhengzhou University, Zhengzhou, Henan 450052, China; 2 Key Laboratory of Cardiac Injury and Repair of Henan Province, Zhengzhou, Henan, China; 3 Henan Province Clinical Research Center for Cardiovascular Diseases, Zhengzhou, Henan 450052, China; 4 Henan Medical Association, Zhengzhou, Henan, China

**Keywords:** hypertrophic cardiomyopathy, N-terminal pro-brain natriuretic peptide, outcomes, predictor

## Abstract

**Background:** Although numerous studies have suggested that elevated N-terminal pro-brain natriuretic peptide (NT-proBNP) is positively correlated with cardiovascular events, especially the heart failure and heart failure-related death (HFRD), evidence of the association between NT-proBNP and the adverse outcomes of hypertrophic cardiomyopathy (HCM) is still relatively limited. The present study was performed to evaluate the relationship between NT-proBNP and outcomes in patients with HCM.

**Methods:** Observational cohort methodology was used in the present study, and a total of 227 patients were included. And the patients were followed for 44.97 ± 16.37 months. Patients were categorized into three groups according to these NT-proBNP tertiles: first tertile (≤910 pg/ml, *n*=68), second tertile (913–2141 pg/ml, *n*=68), and third tertile (≥2151 pg/ml, *n*=69). The adverse outcomes of the present study were all-cause death (ACD) and cardiac death (CD).

**Results:** According to the risk category of NT-proBNP, the incidence of ACD (*P*=0.005) and CD (*P*=0.032) among the three groups showed significant differences. Multivariate Cox regression analysis suggested that the ACD and CD in the third tertile have 7.022 folds (hazard risk [HR] = 7.022 [95% confidence interval [CI]: 1.397–35.282], *P*=0.018) and 7.129 folds (HR = 7.129 [95% CI: 1.329–38.237], *P*=0.022) increased risks as compared with those in the first tertile. Kaplan–Meier survival analyses showed that the cumulative risks of ACD and CD in patients with HCM tended to increase.

**Conclusion:** The present study indicated NT-proBNP was a novel biomarker suitable for predicting adverse prognosis in patients with HCM, which may be used for early recognition and risk stratification.

## Introduction

Hypertrophic cardiomyopathy (HCM) is the most common monogenic cardiovascular disease. It has a disease prevalence of 0.2–0.5% in the general population [1,2]. The major pathological characteristics in HCM are hypertrophy and disarray among cardiomyocytes, interstitial fibrosis, and small vessel disease of the myocardium [3,4]. The disease threatens people in various age groups, and it can vary in clinical presentation, ranging from asymptomatic status over the lifespan to severe cardiac events such as advanced heart failure, systemic embolic events, stroke, malignant arrhythmic events, and even cardiac death (CD) [5–7]. Therefore, there is an urgent need to establish predictors of prognosis in patients with HCM for risk stratification, prevention of complications, and improvement of outcomes. In recent years, even though risk stratification has already been performed for some factors, and prognosis has been predicted in patients with HCM, the long-term clinical outcomes of patients with HCM have remained largely unpredictable.

The neurohormone pro-brain natriuretic peptide is synthesized and released when cardiac myocytes are exposed to hemodynamic stress [8], and it is further cleaved into N-terminal pro-brain natriuretic peptide (NT-proBNP) by proteolytic enzymes. NT-proBNP has been shown to be an effective and sensitive biomarker in heart failure [9,10] and it is also used in risk stratification of HCM and several cardiovascular disorders [11,12].

However, the data about the prognostic impact of NT-proBNP on Chinese patients with HCM are very limited. Therefore, the present study aimed to explore whether the NT-proBNP was an effective and sensitive biomarker suitable for predicting adverse outcomes of these patients.

## Methods

### Study design and population

**Figure 1:**
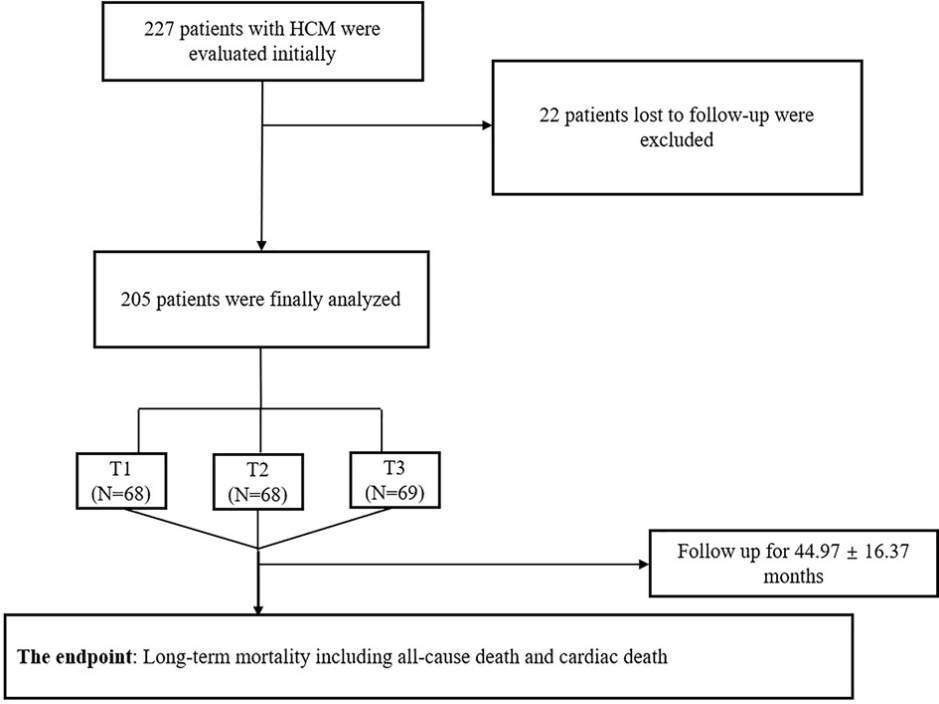
The flowchart of patients’ enrollment

Patients with HCM hospitalized at the First Affiliated Hospital of Zhengzhou University from 2014 to 2018 were included in the present study. Twenty-two patients with HCM were excluded from the study due to loss to follow-up, and the final population was 205 (Figure 1). HCM was diagnosed by a maximum left ventricular wall thickness ≥ 15 mm (or ≥13 mm for patients with electrocardiogram abnormalities or a family history) in one or more left ventricular segments as indicated by echocardiography relying on the 2014 guidelines released by the European Society of Cardiology [13]. The present study excluded patients who had uncontrolled hypertension, cardiac amyloidosis, athlete’s heart, significant coronary artery disease, infection, malignant tumors, liver dysfunction, or renal insufficiency.

### Definitions

Hypertension was defined as any history of hypertension or blood pressure measuring ≥140/90 mmHg on at least three resting measurements at least two different times [14]. Diabetes mellitus was defined as fasting plasma glucose levels up to 7.1 mmol/l or a 2-h post-load glucose concentration of 11.1 mmol/l on multiple measurements, or any receipt of treatment with glucose-lowering drugs [15]. Smoking was defined as any regular cigarette consumption in the previous 6 months, and alcohol consumption was considered as any consumption of alcohol in the past 6 months [16].

### Demographic, laboratory, imaging, and clinical characteristics

Fasting blood samples were obtained for all of the enrolled patients, and the laboratory measurements were performed within 24 h. Data about demographic and clinical characteristics, including age, sex, medical history, body mass index (BMI), heart function classification of New York Heart Association (NYHA), history of hypertension, family history of HCM, and implantable cardiac defibrillator (ICD) implantation, were obtained from medical records. Imaging and laboratory data were also carefully noted. These included white blood cell (WBC), fasting blood glucose, data on renal function, lipid parameters, 12-lead electrocardiography, and echocardiography. During the duration of follow-up, the use of β-blockers, statins, aspirins, diuretics, trimetazidine, amiodarone, and calcium channel blockers was carefully recorded.

### Endpoint

The endpoint was defined as long-term mortality which included all-cause death (ACD) and CD. The CD included sudden cardiac death (SCD) and heart failure-related death (HFRD) in the present study. The definition of SCD was any sudden and unexpected death with or without documented ventricular fibrillation following the new symptoms within 1 h or any death at night without any history of aggravation of symptoms [17]. HFRD was defined as any symptoms of heart failure > 1 h before death.

### Follow-up

All of the enrolled patients underwent regular clinic visits or telephone interviews. The mean follow-up time was 44.97 ± 16.37 months. During this period, all relevant events were carefully assessed and confirmed by trained clinical physicians.

### Statistical analysis

All of the data were analyzed with SPSS 24.0 (SPSS Inc, Chicago, Illinois, United States). Continuous variables are shown as the mean ± standard deviation (mean ± SD) and compared by one-way ANOVA (for data complying with the normal distribution) or Mann–Whitney U test or Kruskal–Wallis variance analysis (non-normally distributed variables). Categorical data are here described as frequencies and percentages and compared using the chi-square (χ^2^) test. NT-proBNP was categorized into three groups by tertile ≤ 910, 913-2141, and ≥ 2151 pg/ml). The cumulative incidence rates of adverse outcomes are shown using Kaplan–Meier curves and were compared using the log-rank test. Variables that showed a significant difference (*P*<0.05) in univariate Cox regression analysis were further entered into multivariate Cox regression analyses which were used to evaluate independent parameters for prognosis. HRs and 95% CIs were calculated. Receiver operating characteristic curves (ROC) were performed to evaluate the accuracy of NT-proBNP in the prediction of outcomes. The *P*<0.05 was considered to be significant.

## Results

### Baseline characteristics

The 205 patients with HCM were categorized into three groups based on the tertile of NT-proBNP: first tertile (NT-proBNP ≤ 910 pg/ml; *n*=68), second tertile (NT-proBNP ≥ 913–2141 pg/ml; *n*=68), and third tertile (NT-proBNP ≥ 2151 pg/ml; *n*=69). As shown in Table 1, the mean age was 55.33 ± 15.00 years at enrollment, and 63.9% were men. In the total population, significant differences among these three groups were found for several variables, including gender, atrial fibrillation, BMI, heart rate, maximal wall thickness (MWT), NYHA III or IV, left ventricular ejection fraction (LVEF), blood urea nitrogen (BUN), left atrial (LA) diameter, hemoglobin, and uric acid (UA) (all *P*-values <0.05). Meanwhile, the other variables, such as age, hypertension, diabetes, triglyceride (TG), creatinine (Cr), smoking, stroke, high-density lipoprotein (HDL), family history, total cholesterol (TC), ICD implantation, estimated glomerular filtration rate (eGFR), low-density lipoprotein (LDL), and left ventricular end-diastolic volume (LVEDV), were not significantly different among these groups (*P*≥0.05).

**Table 1:** Baseline characteristics

Variable	All patients (*n*=205)	First tertile (≤910 pg/ml) (*n*=68)	Second tertile (913–2141 pg/ml) (*n*=68)	Third tertile (≥2151 pg/ml) (*n*=69)	*P*-value
Gender (male)	131 (63.9)	52 (76.5)	41 (60.3)	38 (55.1)	**0.025**
Age at enrollment (years)	55.33 ± 15.00	53.06 ± 13.16	56.95 ± 14.14	55.96 ± 17.29	0.292
Hypertension	79 (38.9)	30 (44.8)	29 (43.3)	20 (29.0)	0.113
Diabetes	18 (8.8)	7 (10.3)	6 (8.8)	5 (7.2)	0.820
Smoking	50 (24.4)	21 (30.9)	14 (20.6)	15 (21.7)	0.309
Alcohol drinking	24 (11.7)	13 (19.1)	4 (5.9)	7 (10.1)	0.050
BMI (kg/m^2^)	24.45 ± 3.61	25.12 ± 3.01	24.62 ± 2.89	23.63 ± 4.56	**0.048**
Atrial fibrillation	20 (9.8)	3 (4.4)	4 (5.9)	13 (18.8)	**0.007**
Stroke	15 (7.3)	4 (5.9)	7 (10.3)	4 (5.8)	0.569
Family history	36 (17.6)	8 (11.8)	16 (23.5)	12 (17.4)	0.197
NYHA III or IV	34 (16.6)	6 (8.8)	10 (14.7)	18 (26.1)	**0.022**
Chest pain	89 (43.4)	28 (41.2)	31 (45.6)	30 (43.5)	0.874
Palpitation	103 (50.2)	34 (50.0)	38 (55.9)	31 (44.9)	0.439
Heart rate (beats/min)	75.35 ± 16.28	74.02 ± 12.74	72.51 ± 14.84	79.42 ± 19.72	**0.032**
Family history of SCD	19 (9.3)	7 (10.3)	4 (5.9)	8 (11.6)	0.483
Unexplained syncope	22 (10.7)	6 (8.8)	12 (17.6)	4 (5.8)	0.067
Resting LVOT obstruction	97 (47.3)	31 (45.6)	38 (55.9)	28 (40.6)	0.188
LVEDV (ml)	91.88 ± 33.48	98.91 ± 31.99	90.37 ± 26.00	86.62 ± 39.94	0.111
MWT (mm)	19.72 ± 4.61	18.39 ± 3.77	19.62 ± 4.48	21.06 ± 5.11	**0.004**
LVEF (%)	63.03 ± 8.72	65.08 ± 5.76	64.11 ± 5.92	60.09 ± 11.96	**0.002**
LA diameter (mm)	40.20 ± 7.17	38.90 ± 5.51	39.57 ± 6.34	41.94 ± 8.77	**0.046**
Hemoglobin (g/l)	135.83 ± 17.97	139.64± 16.20	136.22 ± 17.08	131.70 ± 19.77	**0.034**
WBC (×10^9^)	7.04 ± 2.58	6.88 ± 2.21	6.87 ± 2.56	7.36 ± 2.93	0.457
Neutrophil (×10^9^)	4.46 ± 2.18	4.14 ± 1.99	4.54 ± 2.31	4.71 ± 2.21	0.302
Lymphocyte (×10^9^)	1.92 ± 0.91	2.03 ± 0.69	1.78 ± 0.67	1.94 ± 1.23	0.282
BUN (mmol/l)	6.21 ± 3.20	5.81 ± 1.44	5.76 ± 1.57	7.05 ± 5.04	**0.028**
UA (μmol/l)	351.52 ± 109.05	357.74 ± 92.84	322.16 ± 91.65	374.68 ± 132.50	**0.016**
Cr (μmol/l)	74.36± 27.19	71.71 ± 18.11	71.61 ± 17.06	79.74 ± 39.70	0.135
eGFR (ml/min/1.73 m^2^)	94.60 ± 22.95	98.23 ± 15.12	95.31 ± 24.01	90.27 ± 27.42	0.192
Glucose (mmol/l)	4.87 ± 1.67	4.85 ± 1.03	4.69 ± 1.27	5.10 ± 2.45	0.408
TG (mmol/l)	1.46 ± 1.35	1.72 ± 1.72	1.32 ± 0.63	1.32 ± 1.39	0.139
TC (mmol/l)	3.90 ± 1.04	3.96 ± 1.17	4.04 ± 1.00	3.71 ± 0.93	0.180
HDL (mmol/l)	1.11 ± 0.27	1.07 ± 0.22	1.15 ± 0.30	1.11 ± 0.28	0.274
LDL (mmol/l)	2.55 ± 2.15	2.47 ± 0.79	2.44 ± 0.74	2.73 ± 3.60	0.695
β-blockers	153 (74.6)	48 (70.6)	52 (76.5)	53 (76.8)	0.643
Calcium channel blockers	49 (23.9)	19 (27.9)	14 (20.6)	16 (23.2)	0.595
Aspirin	51 (24.9)	21 (30.9)	18 (26.5)	12 (17.4)	0.176
Statins	74 (36.1)	31 (45.6)	21 (30.9)	22 (31.9)	0.079
Trimetazidine	39 (19.0)	13 (19.1)	15 (22.1)	11 (15.9)	0.660
Amiodarone	8 (3.9)	0 (0.0)	4 (5.9)	4 (5.8)	0.132
Alcohol septal ablation	1 (0.5)	0 (0.0)	1 (1.5)	0 (0.0)	0.663
Surgical septal myectomy	15 (7.3)	3 (4.4)	6 (8.8)	6 (8.7)	0.630
ICD implantation	6 (2.9)	1 (1.5)	2 (2.9)	3 (4.3)	0.873

All the continuous variables are shown as the mean ± SD, and categorical data are described as frequencies and percentages. The bold *P*-values are statistically different.

### Outcomes

As presented in Table 2, 20 cases of ACD were recorded during the follow-up. In total, the incidence of ACD in the first tertile was 2 (2.9%), in the second tertile was 5 (7.4%), and in the third tertile was 13 (18.8%), which was significantly different (*P*=0.005). CD occurred in 16 patients: 2 (2.9%) in the first tertile, 4 (5.9%) in the second tertile, and 10 (14.5%) in the third tertile. The incidence of CD differed significantly among these three groups (*P*=0.032), while the separate incidence of SCD and HFRD showed no significant differences (*P*>0.05).

**Table 2:** Clinical outcomes among three groups

Variable	All patients (*n*=205)	First tertile (≤910 pg/ml) (*n*=68)	Second tertile (913–2141 pg/ml) (*n*=68)	Third tertile (≥2151 pg/ml) (*n*=69)	*P*-value
ACD	20 (9.8)	2 (2.9)	5 (7.4)	13 (18.8)	**0.005**
CD	16 (7.8)	2 (2.9)	4 (5.9)	10 (14.5)	**0.032**
SCD	15 (7.3)	2 (2.9)	4 (5.9)	9 (13.0)	0.078
HFRD	1 (0.5)	0 (0.0)	0 (0.0)	1 (1.4)	1.000

All the continuous variables are shown as the mean ± SD, and categorical data are described as frequencies and percentages. The bold *P*-values are statistically different.

**Figure 2:**
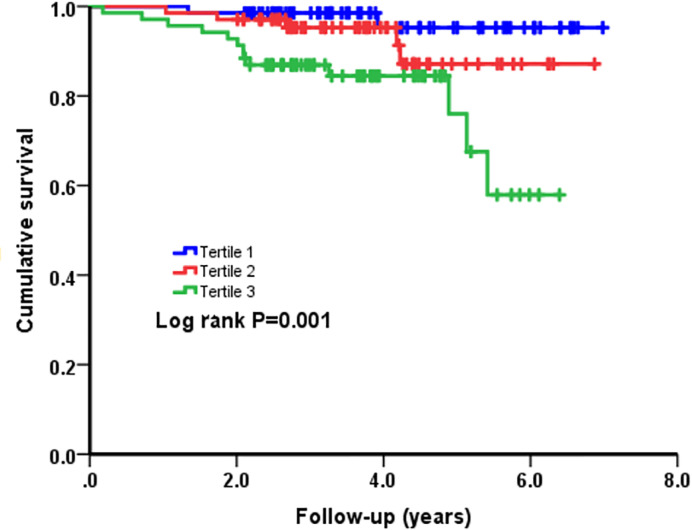
Cumulative Kaplan–Meier estimates of the time to the first adjudicated occurrence of ACD

**Figure 3:**
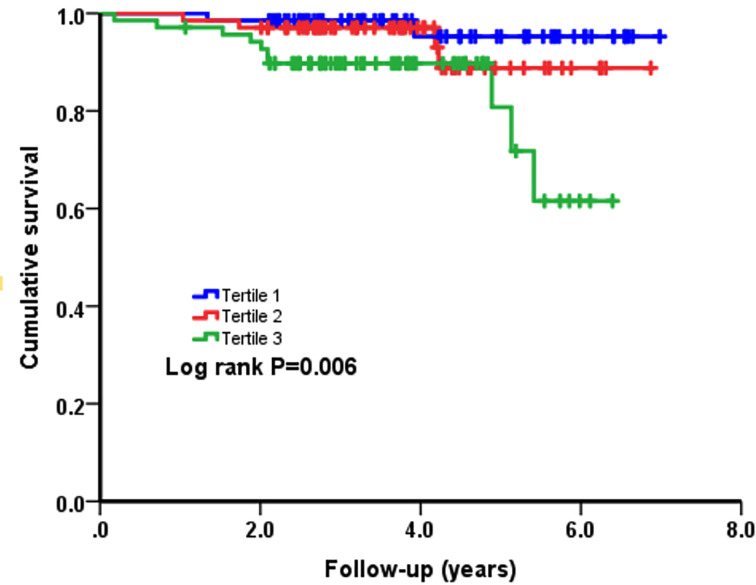
Cumulative Kaplan–Meier estimates of the time to the first adjudicated occurrence of CD

Next, as presented in Figures 2 and 3, Kaplan–Meier curves for NT-proBNP divided by tertiles and ACD and CD were performed. Patients in the third tertile with an NT-proBNP ≥ 2151 pg/ml showed a significantly higher risk of ACD and CD (Log-Rank *P*=0.001 and *P*=0.006, respectively) compared with patients in the first tertile, which was used as the reference, with an NT-proBNP no more than 910 pg/ml.

Univariate Cox regression analysis for predictor variables was conducted. Variables that showed a significant difference (*P*<0.05) were further entered into multivariate Cox regression analysis, which was conducted to evaluate the correlation between NT-proBNP and long-term mortality after adjusting for gender, atrial fibrillation, heart rate, MWT, NYHA III or IV, LVEF, BUN, and BMI. According to the results of multivariate Cox regression analyses, the ACD and CD in the third tertile have 7.022 folds (hazard risk [HR] = 7.022 [95% confidence interval [CI]: 1.397–35.282], *P*=0.018) and 7.129 folds ([HR] = 7.129 [95% [CI]: 1.329–38.237], *P*=0.022) increased risk as compared with those in the first tertile (Tables 3 and 4). Therefore, the higher NT-proBNP had an independently predictive value for adverse outcomes in patients with HCM.

**Table 3:** Cox regression analysis results for ACD

Variables	B	SE	Wald	*P*	HR	95% CI
Gender (male)	0.896	0.507	3.121	0.077	2.450	0.907–6.621
Atrial fibrillation	−0.172	0.843	0.042	0.838	0.842	0.161–4.398
Heart rate (beats/min)	0.005	0.011	0.196	0.658	1.005	0.983–1.027
MWT (mm)	−0.048	0.048	1.001	0.317	0.953	0.868–1.047
NYHA III or IV	−1.887	1.085	3.024	0.082	0.152	0.018–1.271
LVEF (%)	−0.028	0.021	1.787	0.181	0.973	0.934–1.013
BUN (mmol/l)	0.010	0.065	0.023	0.879	1.010	0.889–1.148
BMI (kg/m^2^)	−0.050	0.072	0.477	0.490	0.951	0.826–1.096
NT-proBNP (≤910 pg/ml as reference)			6.629	0.036		
913–2141 pg/ml	0.946	0.849	1.240	0.266	2.575	0.487–13.605
≥2151 pg/ml	1.949	0.824	5.599	0.018	7.022	1.397–35.282

**Table 4:** Cox regression analysis results for CD

Variables	B	SE	Wald	*P*	HR	95% CI
Gender (male)	0.546	0.563	0.940	0.332	1.726	0.572–5.207
Atrial fibrillation	−0.766	1.146	0.447	0.504	0.465	0.049–4.396
Heart rate (beats/min)	−0.001	0.012	0.005	0.946	0.999	0.976–1.023
MWT (mm)	−0.088	0.059	2.245	0.134	0.916	0.816–1.027
NYHA III or IV	−1.632	1.106	2.177	0.140	0.196	0.022–1.709
LVEF (%)	−0.024	0.023	1.060	0.303	0.976	0.933–1.022
BUN (mmol/l)	<0.001	0.092	<0.001	0.999	1.000	0.835–1.197
BMI (kg/m^2^)	−0.094	0.084	1.234	0.267	0.911	0.772–1.074
NT-proBNP (≤910 pg/ml as reference)			6.148	0.046		
913–2141 pg/ml	0.825	0.884	0.872	0.351	2.283	0.404–12.912
≥2151 pg/ml	1.964	0.857	5.253	0.022	7.129	1.329–38.237

**Figure 4:**
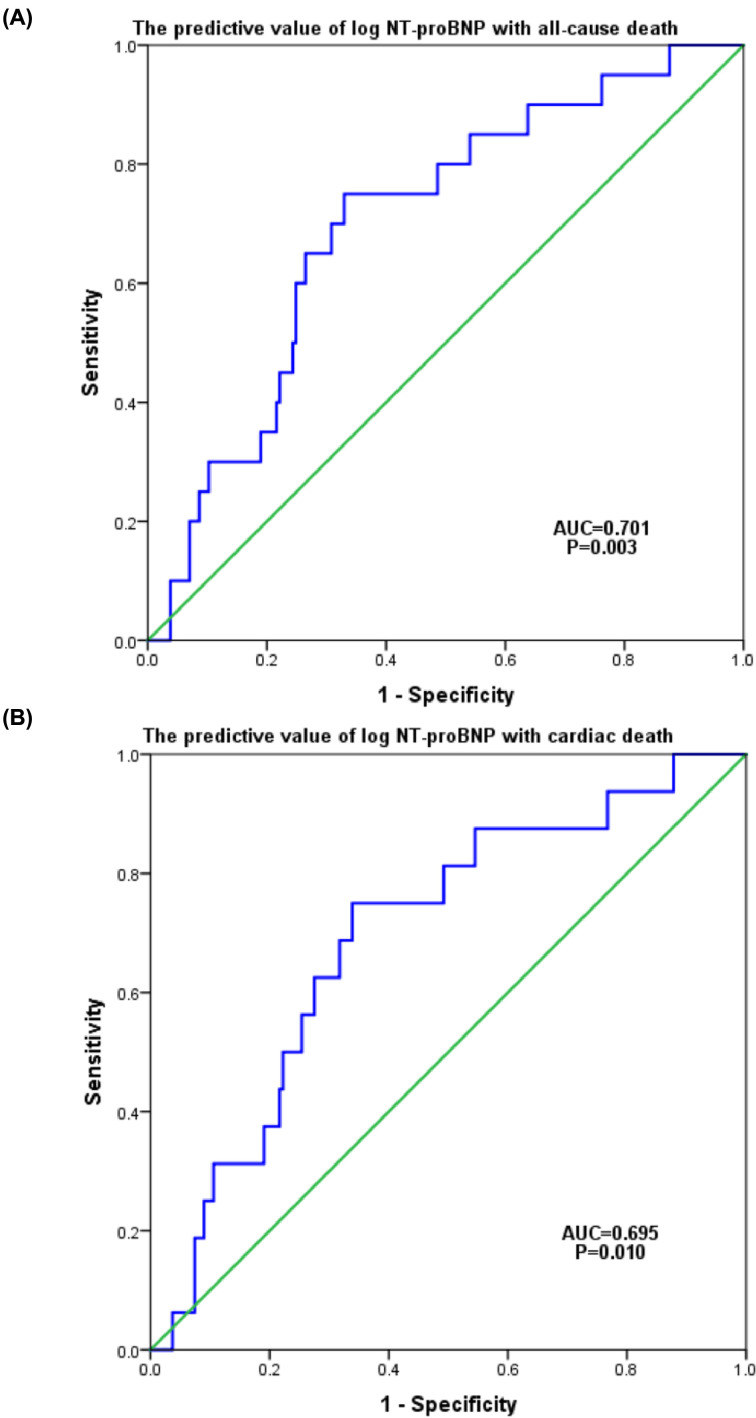
Receiver operating curves of log NT-proBNP levels for outcomes in patients with HCM (**A**) The predictive value of log NT-proBNP with ACD. (**B**) The predictive value of log NT-proBNP with CD.

In ROC curves, a log NT-proBNP cut-off value of 3.30 (NT-proBNP 1993 pg/ml) predicted ACD events with 75.0% sensitivity and 67.0% specificity, and a log NT-proBNP cut-off value of 3.30 (NT-proBNP 1993 pg/ml) predicted ACD events with 75.0% sensitivity and 66.1% specificity (Figure 4A, B).

## Discussion

The present study explored the value of NT-proBNP in predicting long-term outcomes in patients with HCM and suggested NT-proBNP was an independent predictor of long-term mortality. The ACD and CD in the third tertile have 7.022- and 7.129-folds increased risks as compared with those in the first tertile. To decrease the influence of confounding factors, a comprehensive list of characteristics that affected the risk of endpoint was adjusted to assess the correlation between NT-proBNP and clinical outcomes.

Although various novel biomarkers, such as those involving genetic variation [18] and miRNA [19], have emerged recently, their predictive values in the prognosis of patients with HCM are limited. Detection methods for these biomarkers are too complex and expensive to meet the needs of clinical work. Recently, the predictive value of hematologic biomarkers in patients with HCM has drawn increasing attention. These biomarkers include high-sensitivity C-reactive protein [20], monocyte to HDL-cholesterol ratio [21], red blood cell distribution width [22] and mid-regional proatrial natriuretic peptide [23], all of which are available and novel biomarkers in predicting clinical prognosis in patients with HCM.

However, extremely limited data on the prognostic impact of NT-proBNP on patients with HCM have been explored. NT-proBNP is released when cardiac myocytes are exposed to hemodynamic stress [8] and has a strong association with left ventricular hypertrophy and insufficiency [9,24]. Previous studies have revealed that elevated plasma concentration of NT-proBNP in patients with HCM and found that there was a correlation with more left ventricular outflow tract (LVOT) obstruction, worsening symptoms, and reduced exercise tolerance [23,25]. Although the correlation of elevated NT-proBNP value with more risk of adverse clinical events in patients with HCM has been demonstrated by previous studies [11,25,26], while the present study showed differences in the predictive values of NT-proBNP in patients with HCM, which are due to the highly heterogeneous clinical pattern and the differences in enrolled populations.

In the present study, 205 patients with HCM were finally included. These patients were categorized into three groups depending on the tertile of NT-proBNP. The results suggested that patients in the third tertile had higher incidence of ACD and CD compared with those in the first tertile. Although crossing and overlapping of survival curves massively weakens the worth in HRs, Kaplan–Meier survival analyses still showed that the cumulative risks of ACD and CD in patients with HCM tended to significantly increase. Among the three groups, there were significant differences in some baseline characteristic variables, including gender, alcohol drinking, BMI, atrial fibrillation, heart rate, MWT, NYHA III or IV, LVEF, LA diameter, hemoglobin, BUN, and UA. Considering the impact of these confounding factors and some traditional clinical prognostic factors, multivariable regression analyses were performed. The results showed NT-proBNP remained an independent predictor of ACD and CD after adjusting for these confounders. In addition, the results were consistent with a previous report [11]. Thus, the results are valuable and cannot be accidental. However, no significant differences were observed in the incidence of SCD and HFRD in the present study, which is different from previous results [11,26]. And compared with previous studies [11,27], higher mortality was observed in the present study. The differences may result from as followed: First, our study enrolled more patients with severe heart failure (NYHA III or IV) and more patients with resting LVOT obstruction; second, due to the relatively backward economy of our region, the compliance of patients is poor. Third, the rate of ICD therapy was lower in our study than that in previous study [21]. Though previous studies have demonstrated that NT-proBNP is an established biomarker in the patients with HCM, the data on Chinese patients are very limited and our results may be a perfect supplement to predict the outcomes in patients with HCM.

The limitations of the study can be summarized as follows: First, the number of enrolled patients was small, which might have influenced the reliability of the results. Second, this was a single-center retrospective study only including native Chinese patients. Third, the baseline NT-proBNP data were collected only through medical records, which made it difficult to evaluate the effect of dynamic changes in NT-proBNP. In addition, 9.7% of the patients were lost during follow-up, which might introduce biases.

## Conclusion

The present study showed baseline NT-proBNP to be a novel, sensitive, reliable, and effective biomarker in predicting long-term outcomes in patients with HCM, which may be used for early recognition and risk stratification in patients with HCM to prevent complications and improve outcomes. In addition, the study may be a perfect supplement to research into the prognostic impact of NT-proBNP on patients with HCM and may offer insights that could guide further research in the future.

## Data Availability

The data will not be shared, because the identified participant information is included in the data.

## Abbreviations

ACD: all-cause death
BMI: body mass index
BUN: blood urea nitrogen
CD: cardiac death
CI: confidence interval
HCM: hypertrophic cardiomyopathy
HDL: high-density lipoprotein
HFRD: heart failure-related death
HR: hazard risk
ICD: implantable cardiac defibrillator
LVEF: left ventricular ejection fraction
LVOT: left ventricular outflow tract
MWT: maximal wall thickness
NT-proBNP: N-terminal pro-brain natriuretic peptide
NYHA: New York Heart Association
ROC: receiver operating characteristic curve
SCD: sudden cardiac death
UA: uric acid
WBC: white blood cell
